# Supplementary material for: Magnetic alignment enhances homing efficiency of hunting dogs
Source: eLife. 2020 Jun 16;9:e55080. doi: 10.7554/eLife.55080 (PMC7297537; doi:10.7554/eLife.55080)
Supplement: Supplementary file 1. — (A) Table 1 Information about dogs used in the study. Owner = initials of owner accompanying dog during walks, Age = age or age-range during the study period, NOUT = number of outbound trajectories, NIN = total number of inbound trajectories, NT = number of inbound returns using a tracking strategy, NS = number of returns using a scouting strategy. (B) Table 2 Factors in the final GLMMs for the dependent variables (in bold). a) probability for N-S alignment (±45°) during the initial inbound segment (i.e. ‘compass run’); b) probability for scouting strategy; c) efficiency of return; d) speed of inbound trajectory; e) inbound track length. (C) Table 3 Effects used in General Linear Mixed Models. (D) Table 4 Length parameters during different phases of the excursion (data from combined strategies are excluded). (E) Table 5 Circular analyses of individual (‘raw’) and grouped means for azimuth A, B and C during scouting and tracking strategies, and when a scouting strategy was used as the second return strategy (tracking used as a second return strategy not shown). Means were calculated by averaging directional headings for each dog, then calculating a grand mean from all individuals. Raw data were calculated by treating each azimuth as an independent bearing. Note that due to the bimodal preference found within individual dogs for azimuth C, these bearings were treated as axial data. See Figures 6–8. (F) Table 6 Axial analyses of azimuth C (=orientation of the compass run) partitioned into four groups to test for an influence of the owner on the orientation of the compass run during scouting strategy returns. Each analysis corresponds to the orientation of the compass run when the owner was located in one of four cardinal compass directions (±45°) relative to the turning point. Therefore, owner positions relative to the turning point are: owner = magnetic ~ north (316°−45°),~east (46°−135°),~south (136°−225°), or ~west (226°−315°). All data are treated as independent be [file elife-55080-supp1.docx]

**Supplementary File 1A**

Table 1. Information about dogs used in the study.

| **ID** | **Name** | **Breed** | **Owner** | **Age**  **(years)** | **Sex** | **N_OUT_** | **N_IN_** | **N_T_** | **N_S_** |
| --- | --- | --- | --- | --- | --- | --- | --- | --- | --- |
| 1 | Hennessy | Borzoi | PN | 2-3 | M | 53 | 54 | 15 | 39 |
| 2 | Amalka | Dachshund Zwerg Rauhhaar | KB | 2-4 | F | 25 | 27 | 24 | 3 |
| 3 | Hurvinek | Dachshund Zwerg Rauhhaar | KB | 4-7 | M | 46 | 49 | 27 | 22 |
| 4 | Kacka | Dachshund Zwerg Rauhhaar | KB | 2-4 | F | 30 | 31 | 22 | 9 |
| 5 | Pecka | Dachshund Zwerg Rauhhaar | KB | 1-2 | F | 14 | 16 | 11 | 5 |
| 6 | Punta | Dachshund Zwerg Rauhhaar | KB | 1-2 | M | 22 | 25 | 18 | 7 |
| 7 | Terezka | Dachshund Zwerg Rauhhaar | KB | 1-2 | F | 32 | 32 | 25 | 7 |
| 8 | Benda | Dachshund Zwerg Rauhhaar | KB | 1 | F | 16 | 18 | 15 | 3 |
| 9 | Vendulka | Dachshund Zwerg Rauhhaar | KB | 10 | F | 17 | 18 | 13 | 5 |
| 10 | Albi | Dachshund Zwerg Rauhhaar | HF | 9-11 | M | 67 | 71 | 23 | 48 |
| 11 | Hugo | Dachshund Zwerg Rauhhaar | HF | 2-4 | M | 47 | 50 | 34 | 16 |
| 12 | Demi | Dachshund Zwerg Rauhhaar | MJ | 4 | F | 24 | 30 | 20 | 10 |
| 13 | Cecil | Dachshund Zwerg Rauhhaar | AM | 4 | M | 43 | 47 | 41 | 6 |
| 14 | Upir | Dachshund Kaninchen Rauh. | HF | 1 | M | 18 | 20 | 18 | 2 |
| 15 | Figy | Dachshund Kaninchen Rauh. | KB | 2-3 | F | 16 | 17 | 13 | 4 |
| 16 | Kuky | Dachshund Kaninchen Rauh. | KB | 5-6 | M | 4 | 5 | 3 | 2 |
| 17 | Bay | Dachshund Standard Langh. | HS | 3 | M | 12 | 12 | 8 | 4 |
| 18 | Gina | Dachshund Standard Langh. | HS | 1 | F | 8 | 8 | 5 | 3 |
| 19 | Safira | Dachshund Standard Langh. | HS | 2 | F | 12 | 14 | 7 | 7 |
| 20 | Jeny | Dachshund Standard Rauh. | TB | 2 | F | 9 | 9 | 7 | 2 |
| 21 | Amala | Dachshund Standard Kurzhaar | HK | 6 | F | 5 | 5 | 0 | 5 |
| 22 | Kara | German Spaniel | TB | 3 | F | 28 | 31 | 20 | 11 |
| 23 | Bessy | Fox Terrier Smooth | JA | 6-7 | F | 9 | 12 | 8 | 4 |
| 24 | Gofi | Fox Terrier Smooth | JA | 2 | F | 32 | 34 | 24 | 10 |
| 25 | Hard | Fox Terrier Smooth | JA | 1 | M | 12 | 14 | 10 | 4 |
| 26 | Sara | Alpine Dachsbracke | MD | 3-4 | F | 4 | 4 | 2 | 2 |
| 27 | Dona | Welsh Terrier | TB | 4 | F | 17 | 19 | 8 | 11 |
|  | Total | | | | | 622 | 672 | 421 | 251 |

**Supplementary File 1B**

Table 2. Factors in the final GLMMs for the dependent variables (in bold).

|  | *Contributing Factors in final models* | *Num DF* | *Den DF* | *F Value* | *Probability / P value* |
| --- | --- | --- | --- | --- | --- |
| a) | **Probability for N-S compass run** | | | | |
|  | Return strategy | 1 | 644 | 51.70 | <0.0001 |
| b) | **Probability for scouting strategy** | | | | |
|  | Alignment behaviour | 1 | 643 | 46.54 | <0.0001 |
|  | Study site familiarity | 1 | 643 | 15.23 | 0.0001 |
| c) | **Efficiency of return** | | | | |
|  | Alignment behaviour | 1 | 246 | 6.47 | 0.0116 |
| d) | **Speed of inbound trajectory** | | | | |
|  | Shoulder height | 1 | 12.5 | 72.48 | <0.0001 |
|  | Forest path used during inbound return | 1 | 666 | 4.91 | 0.0270 |
|  | Return strategy | 1 | 671 | 17.58 | <0.0001 |
| e) | **Inbound track length** | | | | |
|  | Shoulder height | 1 | 15.6 | 0.3 | 0.5924 |
|  | Forest path used during inbound return | 1 | 672 | 6.32 | 0.0122 |
|  | Beeline distance between dog and owner | 1 | 662 | 1080.88 | <0.0001 |

**Supplementary File 1C**

Table 3. Effects used in General Linear Mixed Models.

| **Variable** | **Mean** | **Standard error** |
| --- | --- | --- |
| Inbound speed (km per hour) | 8.4 | 0.1 |
| Inbound track length (m) | 351.4 | 11.2 |
| Beeline distance between the dog and his owner (m) | 246.3 | 12.1 |
| Dog age (years) | 4.0 | 0.1 |
| Shoulder height (cm) | 30.1 | 0.7 |
| Homing efficiency index (%) | 159.8 | 2.8 |
| **Categorical effects (classes)** | | |
| Return strategy | Tracking or Scouting | |
| Alignment behaviour | Yes or No | |
| Unfamiliar area | Yes or No | |
| Forest path used during inbound return | Yes or No | |
| Sex of the dog | Male or Female | |
| Breed | 10 levels | |

**Supplementary File 1D**

Table 4. Length parameters during different phases of the excursion (data from combined strategies are excluded).

| **Return strategy** | **Part of the excursion** | **Mean** | **Standard error** |
| --- | --- | --- | --- |
| **Tracking**  **N=399** | Outbound trajectory (m) | 384.2 | 16.2 |
|  | Turning trajectory (m) | 76.9 | 3.1 |
|  | Inbound trajectory (m) | 312.2 | 13.3 |
|  | Total (m) | 773.8 | 30. 9 |
|  | Straight distance (m) | 231.1 | 8.4 |
|  | Compass run (azimuth C) (m) | 19.8 | 1.4 |
| **Scouting**  **N=223** | Outbound trajectory (m) | 622.0 | 36.4 |
|  | Turning trajectory (m) | 111.4 | 5.6 |
|  | Inbound trajectory (m) | 384.5 | 20.2 |
|  | Total (m) | 1118.1 | 55. 8 |
|  | Straight distance (m) | 278.2 | 12.9 |
|  | Compass run (azimuth C) (m) | 18.1 | 1.4 |

**Supplementary File 1E**

Table 5. Circular analyses of individual (‘raw’) and grouped means for azimuth A, B and C during scouting and tracking strategies, and when a scouting strategy was used as the second return strategy (tracking used as a second return strategy not shown).

| **Return strategies** | **Azimuth** | **n** | **Mean vector**  **(µ) ± 95% CI** | **Length of mv (r)** | **Circular SD** | **Rayleigh test (Z)** | **Rayleigh test (p)** | **Data type** |
| --- | --- | --- | --- | --- | --- | --- | --- | --- |
| **Scouting** | A (raw data) | 251 | 143° | 0.026 | 155° | 0.163 | 0.849 | Angles |
|  | A (means) | 27 | 207° | 0.209 | 101° | 1.179 | 0.310 | Angles |
|  | B (raw data) | 251 | 56° | 0.097 | 124° | 2.383 | 0.092 | Angles |
|  | B (means) | 27 | 30° | 0.123 | 117° | 0.407 | 0.670 | Angles |
|  | C (raw data) | 251 | 175°/355° ± 5° | 0.437 | 37° | 47.824 | < 10^-12^ | Axial |
|  | C (means) | 27 | 176°/356° ± 7° | 0.824 | 18° | 18.330 | 1.15x10^-8^ | Axial |
| **Scouting**  **as second return** | B (raw data) | 28 | 21° | 0.102 | 122° | 0.291 | 0.751 | Angles |
|  | C (raw data) | 28 | 172°/352° ± 19° | 0.381 | 40° | 4.058 | 0.016 | Axial |
| **Tracking** | A (means) | 26 | 232° | 0.160 | 110° | 0.663 | 0.520 | Angles |
|  | B (means) | 26 | 64° | 0.153 | 111° | 0.612 | 0.547 | Angles |
|  | C (raw data) | 421 | 96°/276° | 0.042 | 72° | 0.748 | 0.473 | Axial |
|  | C (means) | 26 | 92°/272° | 0.227 | 49° | 1.342 | 0.263 | Axial |

**Supplementary File 1F**

Table 6. Axial analyses of azimuth C (= orientation of the compass run) partitioned into four groups to test for an influence of the owner on the orientation of the compass run during scouting strategy returns.

| **Magnetic direction of owner relative turning point (± 45°):** | **n** | **Azimuth C mean vector (µ) ± 95% CI** | **Length of m.v. (r)** | **Circular SD** | **Rayleigh test (Z)** | **Rayleigh test (p)** |
| --- | --- | --- | --- | --- | --- | --- |
| **North** | 71 | 179°/359° ± 12° | 0.370 | 40° | 9.738 | 5.90x10^-5^ |
| **East** | 68 | 173°/353° ± 7° | 0.642 | 27° | 28.014 | < 10^-12^ |
| **South** | 59 | 171°/351° ± 12° | 0.392 | 39° | 9.087 | 1.13x10^-4^ |
| **West** | 53 | 177°/357° ± 16° | 0.322 | 43° | 5.508 | 0.004 |
